# Sanadset 650K: Data on Hadith narrators

**DOI:** 10.1016/j.dib.2022.108540

**Published:** 2022-08-17

**Authors:** Mohammed Mghari, Omar Bouras, Abdelaaziz El Hibaoui

**Affiliations:** Abdelmalek Essaâdi University, Faculty of Science, Computer Science Department, P.O. Box. 2121 M'Hannech II, Tetuan, 93030, Morocco

**Keywords:** Sanad of hadith, Chain of narrators, Sanad dataset, Hadith transmitters, Arabic dataset

## Abstract

The chain of narrators (Sanad) plays a vital role in deciding the authenticity of Islamic hadiths. However, the investigation and validation of such Sanad fully depend on scientists (Hadith Scholars). They ordinarily utilize their acquired knowledge, which in this manner needs a critical sum of exertion and time.

Automated Sanad evaluation using machine learning algorithms is the best way to solve this problem. Therefore, a representative Sanad dataset is required.

This paper presents a full hadith dataset which is named *Sanadset* and is made openly accessible for researchers. *Sanadset* corpus contains over 650,986 records collected from 926 historical Arabic books of hadith. This dataset can be used for further investigation and classification of hadiths (Strong/Weak), and narrators (trustworthy/not) using AI techniques, and also it can be used as a linguistic resource tool for Arabic Natural Language Processing.

Our dataset is collected from online Hadith sources using data scraping and web crawling. The main contribution of this dataset is the extraction of narrator chains that were originally present in textual form within Hadith books. Each observation in the dataset contains complete information about a specific hadith, such as (original book, number, Hadith text, Matn, list of narrators, and the number of narrators).

**Specifications Table**

**Table:** 

Subject	Computer Science
Specific subject area	Islamic Hadith, Arabic Natural Language Processing, Hadith corpus.
Type of data	Text files
How the data were acquired	The dataset was collected from Hadith websites (these hadiths originated from ancient Islamic books). We used web scraping with the help of Python libraries such as Beautiful Soup and Selenium to acquire the dataset.
Data format	Raw, Analyzed, Processed
Description of data collection	The collection and processing of the dataset are mainly divided into two steps: First, the hadith text, numbers, and book fields are collected from Hadith websites. Second, the raw text is further processed to separate Hadith components such as Sanad, Matn, and Narrators. The resulting Matn and Sanad fields are stored in separate columns.
Data source location	The dataset is located at: • Institution: Abdelmalek Essaâdi University • City/Town/Region: Tetuan • Country: Morocco
Data accessibility	Data is available in a public repository. Repository name: Mendeley Data Data identification number: 10.17632/5xth87zwb5.4 Direct URL to data: https://data.mendeley.com/datasets/5xth87zwb5/4

## Value of the Data

• Hadith scholars and Arabic NLP researchers are interested in this dataset [1].
• The Dataset it provides the basis for further research on the nature and structure of Hadiths.
• This dataset can be integrated in many mathematical and statistical models, aimed at disseminating knowledge from hadith compositions. Such as:
  a. Predicting narrator-to-narrator relationships.
  b. Understanding how specific narrators affect the authenticity of Islamic Hadiths.
  c. Conceiving narrators who played important roles in spreading Hadiths.
  d. Predicting the era and location of each narrator.
  e. Finding Hadiths transmitted with similar chain of narration.
  f. Engineering incomplete transmission chains.
  g. Identifying narrators who have similar names.
  h. Building narrator graphs.
  i. Extract teachers and students of a given narrator.

## 1 Data Description

Sanadset is represented as a single CSV file with 6 variables:

○ The first variable contains the original diactritized Hadith text where each of its components is tagged as follows:
  • The transmission chain is surrounded with <SANAD> and </SANAD> tags.
  • Every narrator in the Sanad is surrounded with <NAR> and </NAR> tags.
  • We used <MATN> and </MATN> tags to surround the Matn component.
○ The second variable represents the original book from which the hadith was collected.
○ The third variable represents the hadith number as it appears in the original book.
○ The fourth variable represents the Matn part of the Hadith.
○ The fifth variable is stored in as list format which contains the chain of narrators who tell the hadith.
○ The last variable stores the number of narrators in the transmission chain.

In addition to the Sanadset CSV file, the data repository contains:

○ Ten Arabic Hadith samples are stored in the hadith_samples.csv file.
○ The English translated samples are stored in translated_samples.csv file.
○ The originated classical books from where the data is collected are stored in the books.csv file.

*Sanad dataset includes 650,986 complete hadith examples (see* Table 1 *).*

**Table 1:** Shows a Hadith sample in the dataset from “Sahih al-Bukhari” book. First row: The Hadith in Arabic. Second row: The Hadith translation in English. The two Hadith components are Sanad and Matn are tagged with <SANAD> and <MATN> respectively. Furthermore, every narrator in the Sanad is tagged with <NAR> tag.

## 2 Experimental Design, Materials and Methods

Separating different Hadith components from raw text can be only performed with a great deal of effort and time. These raw texts are present in several ancient Islamic Hadiths books. However, nowadays these books are rewritten by volunteers and uploaded in suitable format to electronic websites and libraries. We found that some websites use HyperText Markup Language (HTML) tags to highlight different Hadith compositions (e.g., colors), and for that reason we chose to collect data from websites.

### 2.1 Data collection and preprocessing

**Fig. 1:**
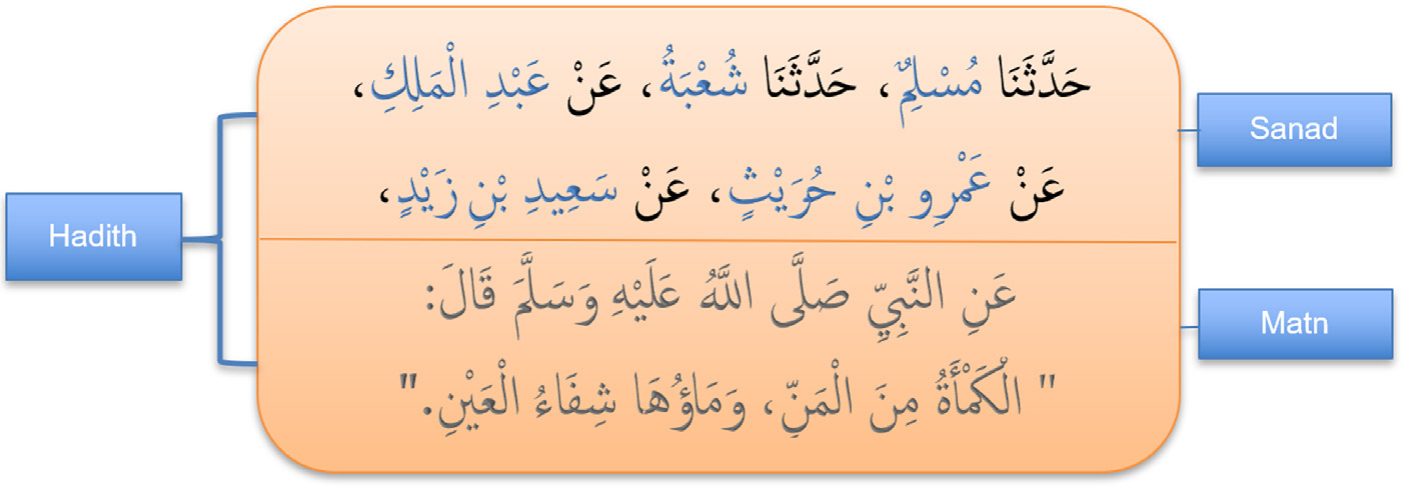
Example of raw Hadith text.

We used data web scrapping technics to collect Hadith data from trusted websites. A total of 650,986 raw reads were initially obtained (see Fig. 1). We constructed a CSV file with three columns; The first column is the raw Hadith text, the second column is the classical book from where the Hadith was rewritten and the third column is the Hadith number.

After collecting the data, we further processed the raw Hadith text field and separate its components with the help of regular expressions and text matching technics (see Fig. 2). The resulting columns are constructed as follows:

- We tagged the Matn component in the raw Hadith text with the corresponding <MATN> tag.
- The transmission chain is tagged with <SANAD> tag.
- Every narrator in the Sanad is tagged with <NAR> tag.
- We found the in some cases hadith writers used words like:  , to identify ambiguous names. We used <IDF> to tag those.
- We isolated Sanad and Matn components and stored them in additional fields.
- We calculated the number of narrators present in every transmission chain and store that number in the Sanad length field.
- We added “No SANAD” text in the Sanad field for hadiths with no narrators.

**2:**
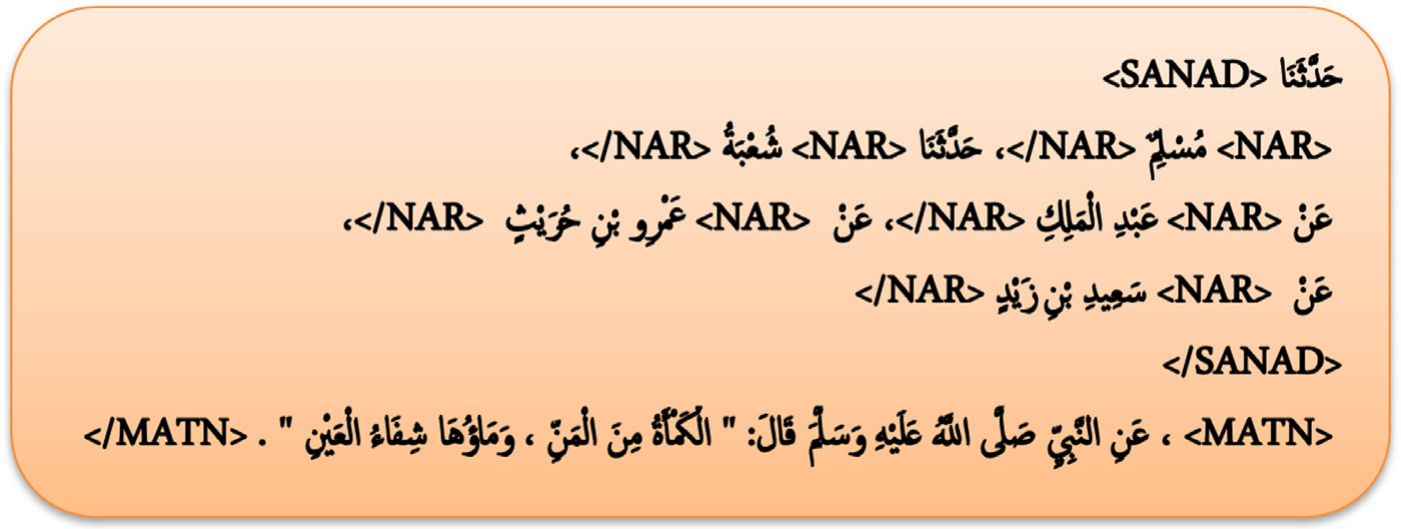
Example of processed Hadith.

### 2.2 Sanadset statistics

*Here are some statistics related to our dataset:*

- The preprocessed resulting dataset contains 650,986 samples.
- Book field contains 926 unique values which means that our dataset is collected from this number of books (find the list of books in the data repository).
- There are 159,558 hadiths with no Sanads.
- 56,053 Hadith contains only one narrator in the transmission chain.
- Some names in the Hadith have different forms even though they refer to the same person. For example, the names .

## Ethics Statements

The authors state that this work involved:

- No human subjects.
- No animal experiments.
- No data collection from social media platforms.

Terms of Use (ToS): Public and free Islamic websites contain hundreds of books in many domains like Hadith. Their aim is to collect books in a text format that can be searched and copied by anyone who is interested in Islamic religion.

Copyright: Data is from public domain, it is dated to decades and centuries. The data does not belong to users on the web resource (i.e., social media). The data is published on free and public Islamic websites and is available to anyone with internet access.

Privacy: While the data is free and public, we anonymize the website and Hadith pages.

Scrapping policies: The web resource does not have any special scrapping policy.

## CRediT authorship contribution statement

**Mohammed Mghari:** Conceptualization, Methodology, Software, Visualization. **Omar Bouras:** Data curation, Writing – original draft. **Abdelaaziz El Hibaoui:** Supervision, Validation, Writing – review & editing.

## Declaration of Competing Interest

The authors declare that they have no known competing financial interests or personal relationships that could have appeared to influence the work reported in this paper.

## Data Availability

Sanadset 650K: Data on Hadith Narrators (Original data) (Mendeley Data).

## Appendix Supplementary materials

Image, application 1

Image, application 2
